# Occurrence of *Echinococcus granulosus* Infection Among Owned Dogs in Kilosa District, Morogoro Region, Tanzania: A Copro‐Antigen ELISA and PCR Analysis

**DOI:** 10.1155/vmi/5116208

**Published:** 2026-10-04

**Authors:** Veronica Emanuel Masawe, Tequeiro Okumu Abuom, Sharon Nthenya Mbindyo, Esron Daniel Karimuribo

**Affiliations:** ^1^ Department of Veterinary Medicine and Public Health College of Veterinary Medicine and Biomedical Sciences Sokoine University of Agriculture P.O. Box 3021 Morogoro Tanzania suanet.ac.tz; ^2^ Department of Clinical Studies Faculty of Veterinary Medicine University of Nairobi P.O. Box 29053-00625 Kangemi Nairobi Kenya uonbi.ac.ke

**Keywords:** copro, copro-prevalence, copro-PCR, dogs, *Echinococcus granulosus*, Tanzania

## Abstract

*Echinococcus* is a taeniid cestode of public health concern with a cosmopolitan distribution that can be transmitted between animals and humans. Cystic echinococcosis caused by *E. granulosus* is among the neglected zoonotic tropical diseases highlighted by the World Health Organization, aiming to diminish the disease burden by 2030. Dogs are the definitive hosts of *E. granulosus* and play a key role in maintaining transmission cycles. Current data on the epidemiology of *E. granulosus* infection in dogs are limited in Tanzania, particularly in the Kilosa District. We conducted a cross‐sectional study between October and December 2025 involving 204 owned dogs in six wards of the Kilosa District. We collected dog fecal samples into a clean Falcon tube (15 mL) and analyzed them by screening with copro‐antigen enzyme‐linked immunosorbent assay (ELISA) and confirmed them by conventional polymerase chain reaction (PCR) with sequencing and BLAST search in GenBank. The overall copro‐prevalence of canine infection was 8.8% (18/204; 95% CI = 5.6–13.6) based on the copro‐antigen ELISA. Further PCR analysis of the 18 ELISA‐positive samples yielded a copro‐PCR prevalence of 16.7% (3/18; 95% CI = 5.8–39.2). Sequencing and BLAST analysis revealed a complete sequence match with *Echinococcus granulosus* clone JKL.4 repeat region (Accession number: KR347168.1) from Mumbai, Maharashtra, India. Only deworming status was a significant predictor of canine infection, with dewormed dogs being 0.321 times (95% CI: 0.114–0.909; *p* = 0.03) less likely to have an infection than undewormed dogs. This is the first report of the presence of *E. granulosus* infection among owned dogs in the Kilosa District. There is a need for the One Health approach in controlling the disease and educating the public on proper protective measures.

## 1. Introduction

*Echinococcus* is a taeniid cestode of public health concern with a cosmopolitan distribution that can be transmitted between animals and humans. All species in the genus *Echinococcus* are suspected and known to be zoonotic, with the complex *E. granulosus sensu lato* (s.l.) and *E. multilocularis* being of major public health importance, causing cystic echinococcosis (CE) and alveolar echinococcosis (AE), respectively [1]. Under contemporary taxonomic frameworks, the *E. granulosus s.l*. complex has been reclassified into five distinct species: *E. granulosus sensu stricto* (s.s.), *E. canadensis*, *E. ortleppi*, *E. equinus*, and *E. felidis*. Within this assembly, *E. granulosus s.s*. (comprising Genotypes G1–G3) is recognized as the predominant etiological agent of human CE, accounting for a disproportionate share of the global socioeconomic and clinical disease burden [2–5]. These genetic differences affect disease severity, control strategies, and the dynamics of transmission.

Dogs are the definitive hosts of *E. granulosus* and play a key role in maintaining transmission cycles, while livestock and wild ruminants serve as intermediate hosts. Humans are the accidental intermediate hosts and acquire infection through ingestion of eggs in contaminated food, water, or soil or through physical contact with an infected dog [6]. Echinococcosis is usually asymptomatic in dogs, despite causing considerable production losses in animals due to liver condemnation, diminished carcass weight, decreased milk yield, sterility, and lowered hide value [7]. In humans, it causes CE and AE due to *E. granulosus s.l.* and *E. multilocularis*, respectively, associated with serious illnesses and even death depending on the cyst location, cyst size, and the involved parasite genotype [8, 9]. Numerous dog‐related risk factors, such as feeding raw offal, free roaming/scavenging, irregular deworming, and the owner’s inadequate health education, have been reported as important causes of canine infection and, as a result, human exposure [10].

An Africa‐wide meta‐analysis study revealed an overall prevalence of canine infection with *E. granulosus* to be 16.9% [11]. In a separate continental review, the aggregated dog prevalence was 24.7% in North Africa, 23.4% in East Africa, and 5.5% in West Africa [12]. Several individual studies on canine infection with *E. granulosus* employing different methodologies have been carried out in many parts of the world [9, 13–22].

Echinococcosis is among the 20 neglected zoonotic diseases targeted for control or elimination by the World Health Organization by 2030 [23]; it is crucial to study the epidemiology of this disease in several geographical settings as a first step toward control. The Kilosa District, noted for its abundant wildlife ecosystem; warm‐humid climate; high free‐roaming dog population; and proximity of wildlife, livestock, and human populations, serves as a pivotal region for investigating echinococcosis. To the best of our knowledge, current data on canine infection in Tanzania, specifically in the Kilosa District, remain limited, hindering the targeted control or eradication of the disease. Therefore, this study aimed to evaluate the prevalence and molecular characteristics of *E. granulosus* in owned dogs located in the Kilosa District, Morogoro Region, Tanzania.

## 2. Materials and Methods

### 2.1. Study Area

This study was conducted in the Kilosa District (see Figure 1), one of the nine district councils of the Morogoro Region located in eastern Tanzania. The district is bordered in the west by the Dodoma Region, to the north by the Gairo District, to the east by the Mvomero District, and to the southeast by the Morogoro Rural District. In the South, the district is bordered by the Iringa Region and the Kilombero District. It lies between latitudes 5°55′ and 7°53′ south and longitudes 36°30′ and 37°30′ east. The Kilosa District has a vast and diverse area, predominantly rural, with scattered villages and a few semiurban centers. The district is semiarid with unimodal rainfall. The livelihood economy is based mainly on agriculture, livestock keeping (cattle, goats, poultry, and dogs), and small‐scale trade [24]. The Kilosa District is characterized by close interaction among humans, livestock, domestic dogs, and wildlife, which may facilitate the transmission of zoonotic pathogens, including *Echinococcus granulosus*, by contributing to maintaining the parasite’s transmission cycle [25]. Furthermore, limited access to veterinary and public health services, weak cross‐sectoral coordination, and logistical challenges, as previously reported in Kilosa District, may increase the risk of zoonotic disease spread [26, 27]. Six wards of the Kilosa District, namely, Mkwatani, Chanzuru, Mabwerebwere, Rudewa, Kimamba, and Magole, were purposively selected as study sites based on recommendations by the district veterinary officer. These recommendations considered predefined epidemiological and operational criteria, including the presence of a substantial dog population and logistical accessibility for field sampling.

**FIGURE 1:**
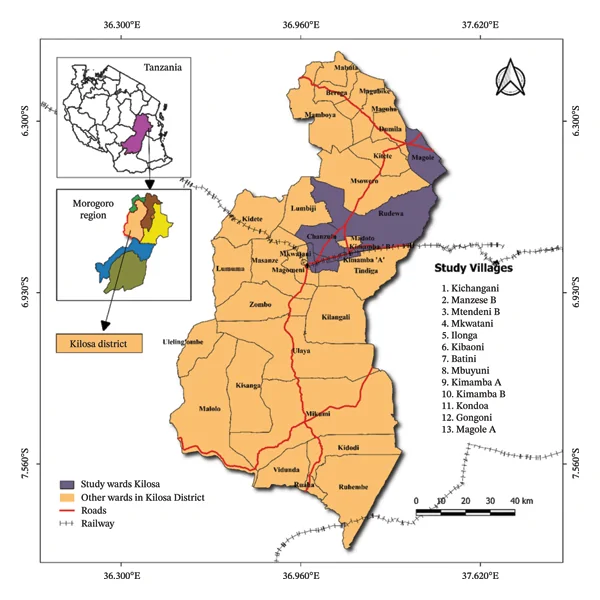
Map of the Kilosa District, Morogoro region, Tanzania, showing the study wards and villages. Map created by using QGIS 3.6 (QGIS development team, Beaverton, OR, USA) based on administrative boundary data.

### 2.2. Study Design and Sampling

A cross‐sectional study design was conducted between October and December 2025 to determine the prevalence of *E. granulosus* infection in dogs and associated risk factors. The sample size of dogs was calculated by using the formula for cross‐sectional studies [28], given as

(1) N=Z2pqd2,

where *N* = desired sample size; *Z* = standard normal deviation at a 95% confidence level (95% CI), usually set at 1.96; *p* = prevalence rate of echinococcosis in dogs in Tanzania, which is 12.4% as previously reported [14]; *d* = allowable error, taken as 5%; and *q* = 1 − *p*. This amounts to a minimum sample size of 167 dogs. Although 204 dogs were sampled and included in this study. Convenience sampling was employed to select households for dog sampling with the assistance of the local livestock field officers. The individual dog was considered as the epidemiological unit of analysis. In the case of more than one dog, proportional sampling of dogs was performed whereby approximately 40% of dogs were sampled, with a maximum of two dogs per household.

### 2.3. Dog Fecal Sample Collection

Dogs were restrained accordingly before fecal sample collection, ensuring the safety of the animal and personnel. Friendly dogs were restrained manually by the dog owner, while aggressive dogs were restrained by using a dog catcher. Approximately 30 g of fecal samples were gently collected from the dog’s rectum using one finger with a gloved hand. In cases where it was not possible to restrain the dog, freshly dropped fecal samples were collected from the floor. The collected sample was put in a clean 15 mL Falcon tube, sealed, labeled, and placed in a cool box before being transported to the College of Veterinary Medicine and Biomedical Sciences laboratory at Sokoine University of Agriculture for further processing. In addition, the animal biodata such as breed, purpose of rearing, age, sex, color, general health status, body condition score, deworming status, presence/absence of live worms, and clinical signs observed at the time of sample collection were recorded digitally for each dog. Furthermore, Global Positioning System (GPS) locations for each household where the dogs were sampled were captured in KoboToolbox.

### 2.4. Copro‐Antigen Enzyme‐Linked Immunosorbent Assay (ELISA) Analysis of Dog Fecal Samples

All fresh fecal samples were subjected to a serological test (copro‐antigen ELISA) by using the copro‐antigen sandwich ELISA kit for *Echinococcus granulosus* from Shenzhen Lvshiyuan Biotechnology Co., Ltd., China, and procedures were followed according to the manufacturer’s instructions with few modifications. The optical density (OD) value was measured immediately with an ELISA reader at a 450 nm wavelength. The assay was valid, as the average OD value of the negative control (NC) was less than 0.20, while the average OD value of the positive control (PC) was greater than 0.5, which is 2.878. Because the average OD value of the PC was too high, the assay was re‐run by diluting the PC into 2x, 3x, and 4x by using a negative sample that had less OD than the NC, all of which showed color change from blue to yellow. The average OD value of the PC at 2x (0.429) was used to calculate the sample‐to‐positive (S/P) ratio value of the samples, and interpretations were made at S/P value < 0.45 as negative and S/P value ≥ 0.45 as positive.

### 2.5. Polymerase Chain Reaction (PCR) Analysis of Dog Fecal Samples

The Quick‐DNATM Miniprep Plus Kit was used for genomic extraction of DNA from the dog fecal samples according to the manufacturer’s instructions. In brief, ≤ 25 mg of the fecal sample was put into a microcentrifuge tube; 95 μL of water, 95 μL of solid tissue buffer, and 10 μL of proteinase K were added; mixed thoroughly; and incubated overnight at 55°C. To remove the insoluble debris, centrifugation at 12,000 rpm for 1 min was performed, and the aqueous supernatant was transferred to a clean microcentrifuge tube. DNA extraction and purification proceeded, and the eluted DNA was stored at −20°C until used in PCR reactions. The concentration of DNA extracted from samples of 2 μL of fecal supernatant was measured by a spectrophotometer (Thermo Fisher Scientific, USA; NanoDrop [ND] 2000). A master mix was prepared with the addition of bovine serum albumin as described previously in order to control the PCR inhibitors available in feces [29]. Conventional PCR was performed targeting the tandem‐repeated DNA sequences of *E. granulosus* (EgG1 *Hae* III) by using one set of primers, which are Eg1121a‐Fwd: 5′‐GAATGCAAGCAGCAGATG‐3′ and Eg1122a‐Rev: 5′‐GAGATGAGTGAGAAGGAGTG‐3′, which produce diagnostic bands at 133 bp [16, 29]. Human cyst DNA and an aliquot of master mix only were used as positive and NCs, respectively. The PCR products were analyzed on a 1.5% Tris‐acetate‐ethylenediaminetetraacetic acid (TAE) agarose gel stained with gel red and visualized under ultraviolet (UV) light (NDR Bio‐Imaging Systems Mini Bis Pro).

### 2.6. Sequencing and BLAST Analysis

To confirm the identity of the amplified PCR products, all 3 positive samples on PCR were sent to Macrogen Europe, Meibergdreef 57, Amsterdam, Noord‐Holland 1105 BA, Netherlands, for DNA sequencing. The obtained forward and reverse sequences were then edited and aligned by using BioEdit software Version 5.0.9 before being BLAST searched in GenBank for similarity.

### 2.7. Statistical Analysis

Data were entered into Microsoft Excel (Version 365, USA), checked for correctness, cleaned, and then exported to IBM Statistical Package for the Social Sciences (SPSS) Version 27 (IBM Corp., Armonk, NY, USA) for statistical analysis. Descriptive statistics such as frequency, mean, median, percentage/proportions, and 95% confidence intervals (95% CIs) were computed and summarized for animal particulars. The prevalence of *Echinococcus granulosus* infection was calculated as the proportion of positive dogs among those tested, with corresponding 95% CIs. Computation of binomial proportion (95% CI) adopted the modified Wald method by using an online calculator (https://www.graphpad.com/quickcalcs/confinterval1/). To identify risk factors associated with canine infection, a binary logistic regression analysis was conducted using the copro‐antigen ELISA results as the dependent variable against animal particulars such as age category, sex, breed, purpose of keeping the dog, color, body condition score, apparent health status, presence of live worms, and deworming status. All risk factors with *p* < 0.25 at univariable logistic regression were further subjected to multivariable logistic regression analysis, as recommended previously [30], to avoid excluding potentially important variables and confounders during the initial screening stage. All three independent variables (breed, age category, and deworming status) were entered simultaneously into the multivariable model using the enter method. Multicollinearity among predictor variables included in the multivariable model was assessed using tolerance and variance inflation factor (VIF) values. A multivariable logistic regression model was then fitted to identify factors independently associated with *E. granulosus* infection. Adjusted odds ratios (AORs) with 95% CIs were reported. Model fit was assessed using the Hosmer–Lemeshow goodness‐of‐fit test. All statistical tests were two‐sided, and statistical significance was set a priori at *p* < 0.05.

## 3. Results

### 3.1. Study Population Characteristics

A total of 204 dogs corresponding to at least one dog from each household were sampled and included in this study, as detailed in Table 1. The median age of sampled dogs was 2 (IQR = 1–3) years. Mkwatani Ward (33.3%) represented the highest proportion of sampled dogs by ward. Local‐breed dogs (95.6%) predominated the sampled dog breeds in the Kilosa District. The sampled dog population comprised 53.9% and 46.1% female and male dogs, respectively. The brown color predominated in the sampled dogs, with more than half of the proportion (60.3%). Approximately half of the sampled dogs had an ideal body condition score (49.5%), with few scoring obese (0.5%). The majority of dogs were kept for security (82.4%) purposes rather than for hunting. Few dogs (6.9%) were apparently sick during sampling.

**TABLE 1:** Particulars of 204 sampled dogs in Kilosa District, Morogoro, Tanzania.

Characteristic	Category	Frequency	%
Ward	Magole	16	7.8
	Chanzuru	8	3.9
	Mabwerebwere	16	7.8
	Kimamba	68	33.3
	Rudewa	42	20.6
	Mkwatani	54	26.5

Breed	Local	195	95.6
	GSD	9	4.4

Age in years	Puppy (< 1 year)	37	18.1
	Young adult (1–3)	122	59.8
	Adult (> 3 years)	45	22.1

Sex	Female	110	53.9
	Male	94	46.1

Color	Brown	123	60.3
	White	18	8.8
	Gray	7	3.4
	Black	16	7.8
	Mixed color	40	19.6

Body condition score	Very thin (1)	4	2.0
	Thin (2)	72	35.3
	Ideal (3)	101	49.5
	Overweight (4)	26	12.7
	Obese (5)	1	0.5

Purpose of keeping dogs	Security	168	82.4
	Hunting	36	17.6

Deworming status	No	46	22.5
	Yes	158	77.5

### 3.2. Copro‐Antigen ELISA Results

The copro‐prevalence of *E. granulosus* infection in dogs by copro‐antigen ELISA was 8.8% (18/204; 95% CI: 5.3–13.6). The locations of sampled households with their results from copro‐antigen ELISA are shown in Figure 2. An equal number (9) of positive males (9.6%) and positive females (8.2%) were observed. Mkwatani Ward (13.0%) had higher copro‐positivity among the wards, followed by Chanzuru, Kimamba, Rudewa, Magole, and Mabwerebwere (12.5%, 7.4%, 7.1%, 6.3%, and 6.3%, respectively). German Shepherd dogs had a higher prevalence (22.2%) compared to local‐breed dogs (8.2%). Puppies had the highest prevalence compared to young adults and adults (18.9%, 6.6%, and 6.7%, respectively). Dogs kept for security purposes had a higher prevalence (9.5%) of infection compared to dogs kept for hunting purposes (5.6%). Mixed‐colored dogs had a higher prevalence (12.5%) than brown‐colored dogs (10.6%), while there were no white, gray, or black copro‐positive dogs. Thin dogs were more positive (15.3%) compared to ideal and overweight dogs (5.0% and 7.7%, respectively) by BCS, whereas there were no copro‐positive dogs with a very thin or obese BCS. About 8.9% of copro‐positive dogs were clinically healthy, while 7.1% of copro‐positive dogs were clinically sick. The majority of copro‐positive dogs had no worms/proglottids seen in feces (9.2%) during sampling, while a few had worms/proglottids seen in feces (5.3%). Higher copro‐prevalence was observed in undewormed dogs (17.4%) than in dewormed dogs (6.3%).

**FIGURE 2:**
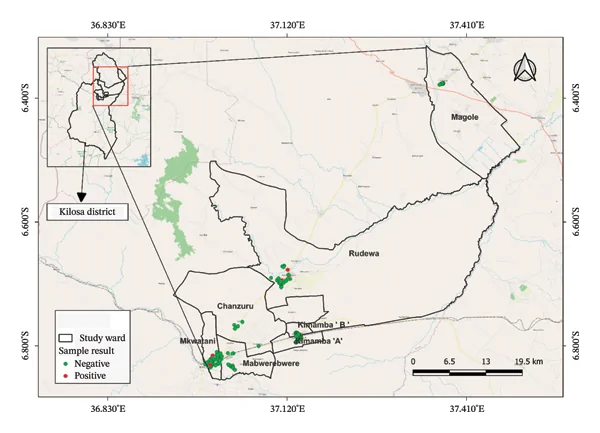
Map of the Kilosa District, Morogoro region, Tanzania, showing the spatial distribution of studied households which are categorized according to the copro‐antigen ELISA results of the sampled dogs. Green color represents households that tested negative, while red color represents households that tested positive. Map created by using QGIS 3.6 (QGIS development team, Beaverton, OR, USA).

### 3.3. Copro‐PCR and Sequencing Analysis Results

Among the 18 copro‐antigen ELISA‐positive samples, only 3 were confirmed to be positive by copro‐PCR (16.7%; 95% CI = 5.8–39.2). Copro‐PCR positivity was confirmed by the presence of a target band size of 133 bp, as seen in Figure 3.

**FIGURE 3:**
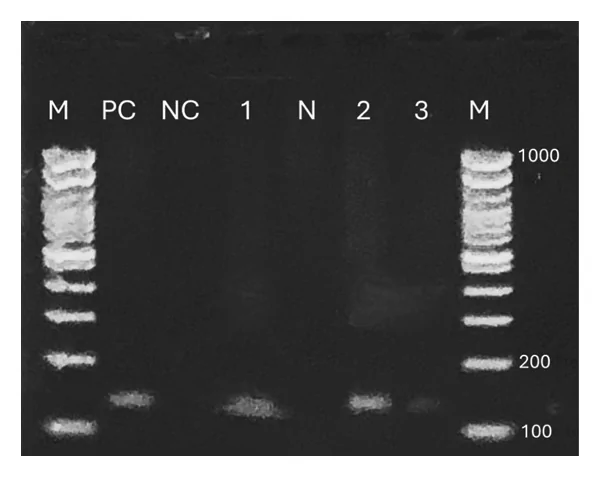
Agarose gel electrophoresis of PCR‐amplified products targeting the *Echinococcus granulosus* repeated tandem DNA sequence. Lane M: 1 kb DNA ladder; Lanes 1–3: positive samples showing the expected 133 bp amplicon; Lane N: negative sample; Lane NC: no‐template negative control, showing no amplification.

Of the three sequenced DNA samples with the same primers that amplified the repeated *Hae*III tandem sequence, one sample showed 100% sequence identity to *Echinococcus granulosus* clone JKL.4 repeat region (Accession number: KR347168.1) from Mumbai, Maharashtra, India. The other two samples had significant background noise, thus low quality, and failed to be sequenced.

### 3.4. Risk Factors Associated With Copro‐Positivity of *E. granulosus* Infection in Dogs

Univariable logistic regression analysis revealed that only deworming status was statistically associated with copro‐positivity at *p* = 0.03 (OR = 0.321, 95% CI: 0.119–0.869), while other parameters were not; see details in Table 2. Dewormed dogs showed lower odds of having infections compared to undewormed dogs.

**TABLE 2:** Summary of univariable logistic regression between animal‐level risk factors and copro‐positivity of *E. granulosus* in dogs.

Variables	Category	*N*	Positive (*n*; %)	OR	95% CI	*p* value
Ward	Magole (Ref)	16	1 (6.3)			0.87
	Chanzuru	8	1 (12.5)	2.143	0.116–39.469	
	Mabwerebwere	16	1 (6.3)	1.000	0.057–17.509	
	Kimamba	68	5 (7.4)	1.190	0.129–10.957	
	Rudewa	42	3 (7.1)	1.154	0.111–11.981	
	Mkwatani	54	7 (13.0)	2.234	0.254–19.653	

Breed	Local (Ref)	195	16 (8.2)			0.17^∗^
	GSD	9	2 (22.2)	3.196	0.612–16.687	

Age category (years)	Puppy (< 1) ‐ Ref	37	7 (18.9)			0.07^∗^
	Young adult (1–3)	122	8 (6.6)	0.301	0.101–0.896	
	Adult (> 3)	45	3 (6.7)	0.306	0.073–1.281	

Sex	Female (Ref)	110	9 (8.2)			0.73
	Male	94	9 (9.6)	1.188	0.451–3.128	

Purpose of keeping	Security (Ref)	168	16 (9.5)			0.45
	Hunting	36	2 (5.6)	0.559	0.123–2.546	

Body condition score	Very thin (Ref)	4	0 (0.0)			0.28
	Thin	72	11 (15.3)	—	—	
	Ideal	101	5 (5.0)	—	—	
	Overweight	26	2 (7.7)	—	—	
	Obese	1	0 (0.0)	—	—	

Apparent health status	Sick (Ref)	14	1 (7.1)			0.82
	Healthy	190	17 (8.9)	1.277	0.157–10.370	

Presence of live worms	No (Ref)	185	17 (9.2)			0.57
	Yes	19	1 (5.3)	0.549	0.069–4.371	

Deworming status	No (Ref)	46	8 (17.4)			0.03^∗∗^
	Yes	158	10 (6.3)	0.321	0.119–0.869	

*Note:* N, number sampled; 95% CI, 95% confidence interval; “—”, OR was not estimated for categories with zero or sparse data due to instability of logistic regression estimates; Ref, reference category.

Abbreviation: OR, odds ratio.

^∗^Not significant, but included in multivariable logistic regression.

^∗∗^Statistically significant and included in multivariable logistic regression.

Multicollinearity assessment indicated no evidence of problematic collinearity among the predictor variables included in the multivariable model, with VIF values ranging from 1.004 to 1.020 and tolerance values ranging from 0.980 to 0.996. In addition, the Hosmer–Lemeshow goodness‐of‐fit test indicated no evidence of poor model fit (*χ* ^2^ = 2.940, df = 4, *p* = 0.57). Of the three variables tested (breed, age category, and deworming status) under the multivariable logistic regression, only deworming status showed a significant statistical association with copro‐positivity of dogs, as shown in Table 3. Specifically, dewormed dogs were 0.321 times (95% CI: 0.114–0.909, *p* = 0.03) less likely to have an infection than undewormed dogs. Although puppies had higher odds of infection than young adults and adult dogs, the association was not statistically significant (*p* > 0.05). German Shepherd dogs were 3.13 times (95% CI: 0.552–17.737, *p* = 0.20) more likely to have an infection than local‐breed dogs, although the association was not statistically significant.

**TABLE 3:** Summary of multivariable logistic regression analysis of potential risk factors associated with copro‐positivity of canine infection.

Parameter category		*B*	S.E.	Wald	df	Sig.	Exp (*B*)	95% CI for EXP (B)	
								Lower	Upper
Breed	GSD	1.141	0.885	1.662	1	0.197	3.130	0.552	17.737

Age category	Puppy (Ref)			3.401	2	0.183			
	Young adult	−0.963	0.576	2.798	1	0.094	0.382	0.124	1.180
	Adult	−1.075	0.748	2.067	1	0.151	0.341	0.079	1.478

Deworming status	Dewormed	−1.135	0.531	4.575	1	0.032	0.321	0.114	0.909

*Note:* Exp(B) = AOR, meaning adjusted odds ratio; 95% CI = 95% confidence interval.

## 4. Discussion

To the best of our knowledge, this is the first report of the existence of *E. granulosus* infection among owned dogs found in the Kilosa District, Morogoro Region, Tanzania. The study reported an 8.8% copro‐antigen ELISA positivity of *E. granulosus* infection among owned dogs in the Kilosa District and a 16.7% copro‐PCR prevalence from the ELISA‐positive samples. Upon sequencing, one sample showed 100% sequence identity to *E. granulosus* clone JKL.4 repeat region from India, while risk factor analysis revealed that dewormed dogs were significantly less likely to have an infection compared to undewormed dogs.

The reported copro‐prevalence is almost equal to the 8.7% prevalence by microscopic examination as reported in Cape Verde [20], but lower than the 12.4% prevalence reported in the Serengeti ecosystem by using the same technique of copro‐antigen ELISA [14]. It is also lower than the 6.4% prevalence of *E. granulosus* infection of free‐roaming, family‐owned dogs reported in three (Rukwa, Katavi, and Arusha) regions of Tanzania by using a modified formol‐ether sedimentation technique [31]. In Africa, the reported prevalence is lower than the 33.5% seroprevalence in Nigeria [9], 12.2% in Uganda by PCR [17], and 26% by copro‐antigen ELISA in Kenya [13]. In addition, in other continents, several studies have reported a higher prevalence of canine infection with *E. granulosus* [15, 18, 21, 32]. In contrast, the reported prevalence is higher than the 2.4% prevalence by necropsy reported in Angola [33]; 1.7% in the Tibet Autonomous Region, China, by using copro‐antigen ELISA [34]; and 5.5% and 6.1% in Nigeria by using multiplex PCR [19] and serum antibody ELISA test kit [35], respectively.

Mkwatani Ward had the highest (13.0%) copro‐positivity among the wards, which could be due to a proportionally large number of sampled dogs. On the other hand, Chanzuru Ward households were very scattered, which explains the reason for the lowest proportion of sampled dogs among the wards. The 95.6% proportion of local breeds among sampled dog breeds shows that the majority of owned dogs in the Kilosa District are local breeds. The higher prevalence observed in German Shepherd dogs compared to local/mongrel dogs could be due to a small sample size; thus, it should be interpreted with caution. Approximately half of the sampled dogs had an ideal body condition score out of 5, meaning that the majority of dogs in the Kilosa District are in good body condition.

The majority of copro‐antigen–positive dogs were apparently healthy, which accounts for the disease not showing obvious clinical signs in definitive dogs, and it is mostly associated with mild clinical signs depicted by some of the sick dogs. The fact that 77.5% of the sampled dogs had been dewormed shows that most dog owners care about their dogs by ensuring regular deworming of their dogs.

The highest prevalence of infection observed in puppies compared to young adults and adults can be attributed to the low immunity of puppies to fight against infection and the acquired immunity or resistance in older adults, as reported previously in Turkana, Kenya [13]. Thin dogs had the highest prevalence of infection compared to ideal and overweight dogs. This can be explained by the fact that poor management of dogs leads to weak dogs with poor body condition scores, which increases their chance of having an infection. In addition, undewormed dogs exhibited a higher prevalence than dewormed dogs, indicating regular deworming is protective against worm infestations. This has also been reported previously, where dewormed dogs and dogs that were kept under confinement had a decreased risk of seropositivity [9].

Dogs in the Kilosa District were mainly kept for either security or hunting purposes. There was a higher prevalence observed in dogs kept for security purposes than those kept for hunting reasons. This is in contrast with the higher seropositivity observed in hunting dogs in southwest Nigeria, although they reported dogs were also used for security purposes [9].

Of the three variables tested (breed, age category, and deworming status) in multivariable logistic regression, only deworming status was identified as a significant predictor of copro‐antigen positivity in the dog population. Specifically, dewormed dogs were 0.321 times less likely to have an infection than undewormed dogs. This aligns with findings from Nigeria and Southern Chile [9, 36]. In contrast, someone else reported deworming as not a significant predictor of canine infection, but rather dogs that were allowed to roam free, fed raw offal, and aged 5 years or below were at relatively high risk of being copro‐antigen–positive [13]. Because the current study did not assess other management practices of dogs, the results should be interpreted cautiously, as deworming alone is unlikely to interrupt *Echinococcus granulosus* transmission in Kilosa District. Therefore, a comprehensive control strategy of CE in Kilosa District should be tailored to a One Health approach that includes regular deworming with praziquantel, responsible dog confinement, ensuring safe disposal or thorough cooking of livestock offal, strengthening meat inspection and slaughter hygiene, and improving community awareness of the parasite’s transmission cycle.

Parasitological detection of adult worms at necropsy or after arecoline purgation has served as a reliable diagnosis for *E. granulosus*, although it is difficult to use in large‐scale epidemiologic studies because it is laborious, biohazardous, and lacks sensitivity. While the microscopic examination of taeniid eggs after fecal sedimentation or flotation techniques has served as a reliable screening method in the dog population, the techniques also have low sensitivity, as they cannot differentiate taeniid eggs to genus or species level. Mass screening with serum or copro‐ELISA has helped as an alternative for mass screening of dog populations with increased sensitivity and specificity. Copro‐antigen ELISA exhibits lower specificity than molecular methods due to the possible cross‐reaction with copro‐antigens from other taeniid cestodes, including *Taenia* species commonly infecting dogs. In addition, copro‐antigen ELISA does not detect parasite DNA; rather, it detects parasite excretory/secretory antigens, which may be present approximately within 1 week postsuccessful anthelmintic treatment, as previously reported [37]. Confirmation with molecular methods has been shown to increase specificity and identification to the species level, especially with the use of repeated tandem sequences as target genes. Further sequencing and genotyping by using mitochondrial markers, such as Cytochrome c oxidase subunit 1 (*cox1*) and NADH dehydrogenase subunit 1 (*nad1*), or whole mitochondrial genome analysis has been performed to characterize the *Echinococcus granulosus* genotypes [18, 20, 22, 38].

The highest OD of the PC is due to the use of experimentally infected dogs and not naturally infested dogs; thus, the assay was optimized by dilution of the PC and using the 2x OD values for PCs.

The copro‐antigen ELISA prevalence of *E. granulosus* in the present study might have been overestimated due to the cross‐reactive nature of the copro‐antigens and the presence of copro‐antigens shortly after effective anthelmintic administration or death of the parasite. Therefore, the results should not be interpreted as definitive evidence of active infection in the absence of molecular confirmation; rather, copro‐antigen ELISA should be interpreted primarily as a sensitive screening method. On the other hand, the low copro‐PCR prevalence can be attributed to the lower sensitivity of copro‐PCR due to intermittent egg shedding, the presence of PCR inhibitors in feces, low DNA concentration, and possible DNA degradation during DNA extraction. Despite this, the use of the EgG1 *Hae* III repeated 133‐base pair target gene has shown efficiency in detecting even a single egg of *E. granulosus* in feces, and the ability to differentiate co‐infection with taeniid eggs [29]. Therefore, there is a high chance that the positive samples on copro‐PCR were solely from *E. granulosus* infection. Further sequencing and BLAST search revealed 100% identical similarity to *Echinococcus granulosus* clone JKL.4 repeat region (Accession number: KR347168.1) from Mumbai, Maharashtra, India. This suggests that not only was the amplified DNA really from the *Echinococcus granulosus* genome, but it also accounts for the higher sensitivity of repeated tandem sequences as a genetic marker for *E. granulosus*, which can be detected in even a single egg count without cross‐amplification with DNA from other taeniids [29]. The complete genome identical to the *E. granulosus* sequence previously reported in India (Accession number: KR347168.1) should not be interpreted as the possible transboundary spread of *E. granulosus* but rather as more likely to reflect a shared genetic lineage or conserved genomic region among *E*. *granulosus* isolates.

Despite the strength of this study in estimating the prevalence of *E. granulosus* and associated risk factors in owned dogs found in Kilosa District, this study has several limitations. (1) The current study provided baseline data regarding owned dogs, leaving out the stray dogs, which might have underestimated the prevalence, as the stray dogs are highly exposed to slaughterhouse wastes, which serve as a source of infection. The prevalence of *E. granulosus* in stray dogs should also be studied in Kilosa District, as the stray dogs pose a greater public health threat due to unrestricted roaming, lack of regular deworming, and environmental contamination with feces. (2) The study wards’ selection was based on recommendations from the DVO, which may hinder generalizability of the results to the entire Kilosa District, necessitating further studies employing district‐wide probability sampling. (3) Although most dogs (173/204; 84.8%) were sampled from different households, a small proportion of dogs originated from the same household and may have shared environmental and management‐related exposures. Household‐level clustering was not explicitly accounted for in the statistical analysis, which may have had a minor effect on the precision of the estimated associations. (4) The study did not include a spiked PC to evaluate DNA extraction efficiency and PCR inhibition in fecal samples; thus, the possibility of false‐negative PCR results cannot be excluded due to inefficient DNA recovery or the presence of inhibitors. (5) Also, the application of copro‐PCR testing targeting the EgG1 *Hae* III repeated 133‐base pair DNA sequence of the *E. granulosus* genome for sequencing purposes has limited sensitivity due to the short region being amplified and should be interpreted as species confirmation only. Consequently, the study could not determine which *E. granulosus sensu lato* genotypes are circulating in Kilosa District, as different genotypes vary in host preference, zoonotic potential, pathogenicity, and transmission ecology.

## 5. Conclusion

The study demonstrates the presence of *E. granulosus* infection among owned dogs for the first time in the Kilosa District. Considering the zoonotic potential of *E. granulosus*, the presence of infection in dogs, who are the definitive hosts, serves as a source of environmental contamination for human exposure. This calls for the “One Health” approach in controlling canine infection, educating dog owners on proper management practices, and community awareness of the proper preventive measures. Since no published data were found in Kilosa District regarding human CE cases, this study recommends a follow‐up study to quantify the human CE burden in Kilosa District so that appropriate One Health evidence‐based interventions can be prioritized to prevent further incidences. Future research should focus on molecular characterization of *Echinococcus* isolates by using mitochondrial markers, such *cox1* and *nad1*, or whole mitochondrial genome analysis to identify circulating genotypes and better understand transmission patterns between dogs, livestock, wildlife, and humans.

## Author Contributions

Veronica Emanuel Masawe conceptualized the idea, designed the project, collected data, performed laboratory work, analyzed the data, and wrote the manuscript draft. Tequeiro Okumu Abuom contributed to the design of the project, supervised the research, and revised the manuscript. Sharon Nthenya Mbindyo and Esron Daniel Karimuribo supervised the research and revised the manuscript. The corresponding author had full access to all of the data in this study and takes complete responsibility for the integrity of the data and the accuracy of the data analysis.

## Funding

This work was funded by the Sokoine University of Agriculture (SUA) through the Higher Education for Economic Transformation (HEET) Project.

## Disclosure

The lead author affirms that this manuscript is an honest, accurate, and transparent account of the study being reported; that no important aspects of the study have been omitted; and that any discrepancies from the study as originally planned have been explained. The funder had no role in the study design; collection, analysis, or interpretation of data; writing of the manuscript; or the decision to submit the manuscript for publication. All authors have read and approved the final version of the manuscript.

## Ethics Statement

The ethical approval was obtained from the University of Nairobi, Faculty of Veterinary Medicine Biosafety, Animal Use and Ethics Committee (BAUEC) with REF: FVM BAUEC/2025/642 and from the Sokoine University of Agriculture, Directorate of Postgraduate Studies, Research, Technology Transfer and Consultancy (DPRTC) with reference number: DPRTC/R/186/55 before the commencement of this study. Permission for field data collection was obtained from the President’s Office for Regional Administration and Local Government (PO‐RALG). Consent for sampling the dogs was obtained from the dog owners before sampling the dogs. Animal welfare was considered during sample collection, ensuring minimal stress to the animal and applying gentle pressure to avoid traumatizing the dog’s rectum. Also, precautions were taken not to transmit diseases from one household to the other by using disinfectants before and after sampling. Furthermore, unused processed fecal samples were incinerated to prevent transmission of potentially infectious agents.

## Consent

Please see the Ethics Statement.

## Conflicts of Interest

The authors declare no conflicts of interest

## Data Availability

The data that support the findings of this study are available from the corresponding author upon reasonable request.
